# Effect of Different Software Programs on the Accuracy of Dental Scanner Using Three-Dimensional Analysis

**DOI:** 10.3390/ijerph18168449

**Published:** 2021-08-10

**Authors:** Keunbada Son, Wan-Sun Lee, Kyu-Bok Lee

**Affiliations:** 1 Department of Dental Science, Graduate School, Kyungpook National University, Daegu 41940, Korea; sonkeunbada@gmail.com; 2 Advanced Dental Device Development Institute (A3DI), Kyungpook National University, Daegu 41940, Korea; 3 Department of Dental Technology, Busan Institute of Science and Technology, 88 Srang-ro, 132 Beon-gil, Buk-gu, Busan 616-737, Korea; ws.lee@bist.ac.kr; 4 Department of Prosthodontics, School of Dentistry, Kyungpook National University, Daegu 41940, Korea

**Keywords:** 3D analysis, 3D comparison, alignment, 3D dental scanner, accuracy, dentistry

## Abstract

This in vitro study aimed to evaluate the 3D analysis for complete arch, half arch, and tooth preparation region by using four analysis software programs. The CAD reference model (CRM; N = 1 per region) and CAD test models (CTMs; N = 20 per software) of complete arch, half arch, and tooth preparation were obtained by using scanners. For both CRM and CTMs, mesh data other than the same area were deleted. For 3D analysis, four analysis software programs (Geomagic control X, GOM Inspect, Cloudcompare, and Materialise 3-matic) were used in the alignment of CRM and CTMs as well as in the 3D comparison. Root mean square (RMS) was regarded as the result of the 3D comparison. One-way analysis of variance and Tukey honestly significant difference tests were performed for statistical comparison of four analysis software programs (α = 0.05). In half-arch and tooth preparation region, the four analysis software programs showed a significant difference in RMS values (*p* < 0.001), but in complete-arch region, no significant difference was found among the four software programs (*p* = 0.139). As the area of the virtual cast for 3D analysis becomes smaller, variable results are obtained depending on the software program used, and the difference in results among software programs are not considered in the 3D analysis for complete-arch region.

## 1. Introduction

From the acquisition of virtual casts in digital dentistry to the dental computer-aided design and computer-aided manufacturing (CAD/CAM) process of dental prostheses, a virtual digital workflow is essential [1,2,3,4]. A digital workflow using three-dimensional (3D) data enables the fabrication of a dental prosthesis that is more visual and accurate than those using a conventional workflow [5]. The conventional workflow for manufacturing dental prostheses requires the fabrication of physical dental casts, and impressions are taken using polyvinyl siloxane materials for teeth and soft tissues [5]. A dental prosthesis can be manufactured directly from a physical dental cast, or a partial digital workflow for manufacturing a dental prosthesis can be performed by acquiring a virtual cast using a desktop scanner [5]. On the contrast, the fully digital workflow directly scans teeth and soft tissues using an intraoral scanner and fabricates a dental prosthesis in a virtual cast without manufacturing a physical dental cast [1,2,3].

Dental prosthesis fabricated by a digital workflow is evaluated for various purposes [6,7,8,9]. Studies have evaluated errors of the virtual cast that occur when acquiring a virtual cast using an intraoral or desktop scanner, and this is considered the most important evaluation factor because virtual cast errors have the greatest influence on the results of manufacturing a dental prosthesis [6,10,11]. To evaluate errors generated in the CAM process, the precision of CAM is evaluated by comparing the virtual crown extracted in the CAD process with the virtual crown scanned after the CAM process [12,13].

Previous studies have proposed a method to evaluate the accuracy of an intraoral or desktop scanner [1,2,3,4,6,7,8,10,11,14,15,16]. The accuracy of the virtual cast was evaluated by measuring the linear distance between specific oral structures or between teeth of dental arches [8,17]. Accuracy of complete-arch impressions is evaluated through linear measurement between specific structures or between teeth; however, accuracy of other half arches or specific areas is evaluated by 3D analysis in previous studies [7,10,11,17]. The point cloud of the object is acquired by using an intraoral or desktop scanner, and a virtual cast is completed by reconstructing the point cloud into a mesh through an algorithm of alignment and merging in the software program [18,19,20,21]. Therefore, a 3D analysis method capable of analyzing the accuracy of all point clouds of a virtual cast is preferred in previous studies [1,11].

**Figure 1:**
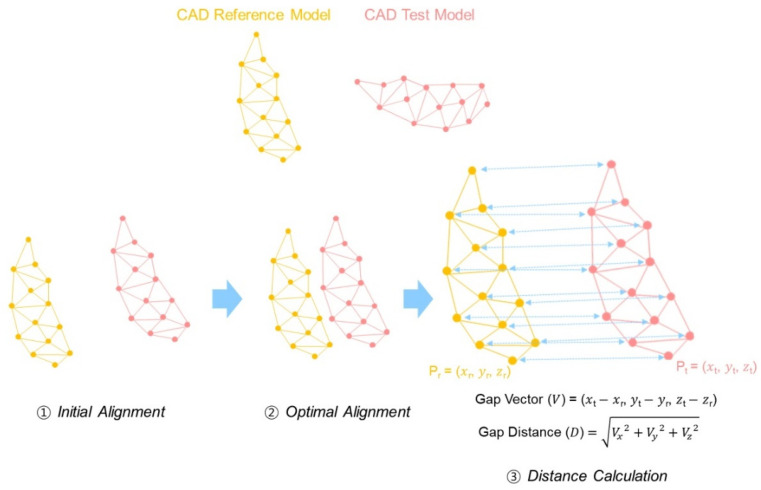
Schematic representation of alignment procedure and distance calculation.

A 3D analysis is possible only in specific analysis software programs, and analysis software programs released by various manufacturers are used [22,23,24,25]. In 3D analysis, the CAD reference model (CRM) and CAD test models (CTMs) are first aligned and 3D comparison is then performed, which is a general 3D analysis process (Figure 1) [26,27,28]. In the alignment process, the approximate positions of CRM and CTMs are initially aligned, followed by optimal alignment (Figure 1). The optimal alignment algorithm uses the iterative closest point (ICP) algorithm to minimize the difference between point clouds [28]. After the alignment of CRM and CTMs, 3D comparison is performed, in which the absolute mean distance between corresponding points with each other is calculated by using the root mean square (RMS) [29,30,31]. The alignment and distance calculation algorithms differ from one software manufacturer to another [29,30]. However, to the best of the authors’ knowledge, no studies have assessed differences in accuracy among analysis software programs.

Therefore, this study aimed to evaluate the difference in accuracy according to the analysis software program used. The null hypotheses are as follows: First, there is no difference in the RMS values of four analysis software programs (Geomagic control X, GOM Inspect, Cloudcompare, and Materialise 3-matic) in three analysis area (complete arch, half arch, and tooth preparations). Second, different alignment methods and different RMS calculation methods for 3D analysis do not affect the RMS value.

## 2. Materials and Methods

**Figure 2:**
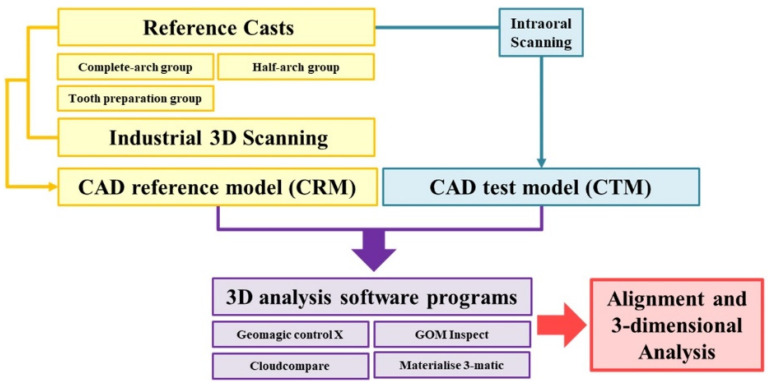
Experimental design.

The sample size of this study was determined by using the power analysis software (G*Power v3.1.9.2; Heinrich-Heine-Universität Düsseldorf, Düsseldorf, Germany) from the results of pilot experiments (N = 5) performed with the same materials and methods as in the present study (N = 20; actual power = 96.51%; power = 96%; α = 0.05). Figure 2 shows the experimental design. The maxillary complete arch of the study model (ANA-4; Frasaco GmbH, Tettnang, Germany) was determined as reference casts (complete-arch group and half-arch group), and the right upper first molar was reduced as a condition for a ceramic crown (finish line, 1-mm wide chamber with a supragingival finish line; occlusal surface and axial wall, reduction of 1.5-mm, convergence angle of 6 degrees; tooth preparation group). CRM was obtained by using an industrial scanner (Solutionix C500; MEDIT, Seoul, Korea) for reference models (N = 1 per region). The industrial scanner used in this study was verified by the manufacturer to have an accuracy of less than 5 µm. For CTMs, reference models were scanned by using an intraoral scanner (CS3600; Carestream, Atlanta, GA, USA) (N = 20 per software program). All experimental procedures were performed by a skilled examiner (K.S.). All obtained CRMs and CTMs were deleted by using the mesh-editing software (Meshmixer; Autodesk, San Rafael, CA, USA); except for the matched area, complete arch (volume, 21,231 mm^3^; surface area, 4177 mm^2^), half arch (volume, 10,048 mm^3^; surface area, 2142 mm^2^), and tooth preparation (volume, 333 mm^3^; surface area, 160 mm^2^) which were modified with CRM and CTMs of the same volume and surface area.

The 3D analysis software program used four different software programs, namely, Geomagic control X (3D Systems, Rock Hill, SC, USA), GOM Inspect (GOM, Braunschweig, Germany), Cloudcompare (Cloudcompare, Paris, France), and Materialise 3-matic (Materialise, Leuven, Belgium) (Table 1). CRM and CTMs were aligned and 3D-compared according to the protocol of each software program by one investigator (K.S.) (Table 1).

Initial alignment was performed to align the approximate position, and optimal alignment was performed to align the minimum distance of each corresponding point cloud of CRM and CTMs (Figure 1). The distances of all corresponding points were calculated (Figure 1), and the results of the 3D comparison were calculated by using the RMS formula.

$$RMS=\frac{1}{\sqrt{n}}\cdot \sqrt{\overset{n}{\underset{i=1}{\sum }}D_{i}{}^{2}}$$

where $D_{i}$ represents the gap distance of point i of CRM and CTM, and n is the number of all points evaluated. The color difference map was set in a color range of ±1.0 mm in each software program, but it was not possible in Materialise 3-matic. Materialise 3-matic does not provide the ability to modify the color map extents, it has automatically adjusted the color map extents for reference only. However, since the calculation and statistical analysis of RMS were possible in Materialise 3-matic, it was not excluded from the present study. The red color region (positive error: +10 µm~+100 µm) indicates that the CTM is located above the CRM, and the blue color region (negative error: −10 µm~−100 µm) indicates that the CTM is located below the CRM. Since the color map means the RMS value, the distribution of the color map was not analyzed statistically, and visual analysis was performed by two investigators (K.S. and W.-S.L.).

Moreover, the identical alignment procedure was performed in Geomagic control X, and RMS calculation was performed in each software program. The alignment procedure was performed in each software program, and the identical RMS calculation was performed in Geomagic control X. Consequently, the effect of different RMS calculation methods and different alignment procedures on the result was evaluated.

Statistical analysis was performed by using a statistical software (IBM SPSS Statistics v25.0; IBM Corp., Armonk, NY) (α = 0.05). All acquired RMS data had a normal distribution. Differences in mean RMS values among each software program group were verified by using a one-way analysis of variance (ANOVA) and Tukey honestly significant difference (HSD) test, and the interaction effect between the 3D analysis software program and the analysis region was verified by using a two-way ANOVA.

## 3. Results

**Figure 3:**
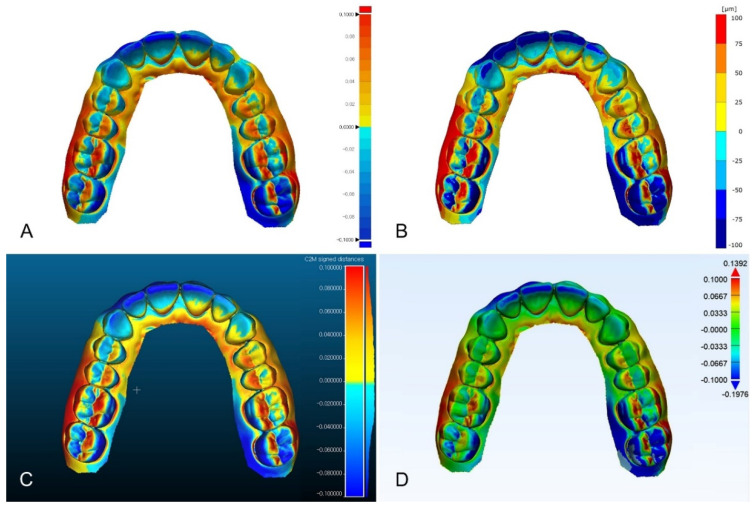
Comparison of color difference maps of complete-arch region evaluated by using four analysis software programs: (**A**) Geomagic control X; (**B**) GOM Inspect; (**C**) Cloudcompare; (**D**) Materialise 3-matic.

**Figure 4:**
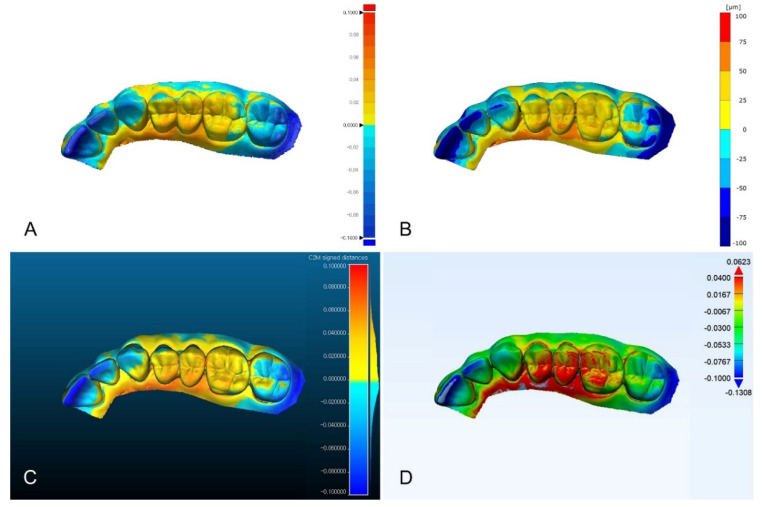
Comparison of color difference maps of half-arch region evaluated by using four analysis software programs. (**A**) Geomagic control X; (**B**) GOM Inspect; (**C**) Cloudcompare; (**D**) Materialise 3-matic.

**Figure 5:**
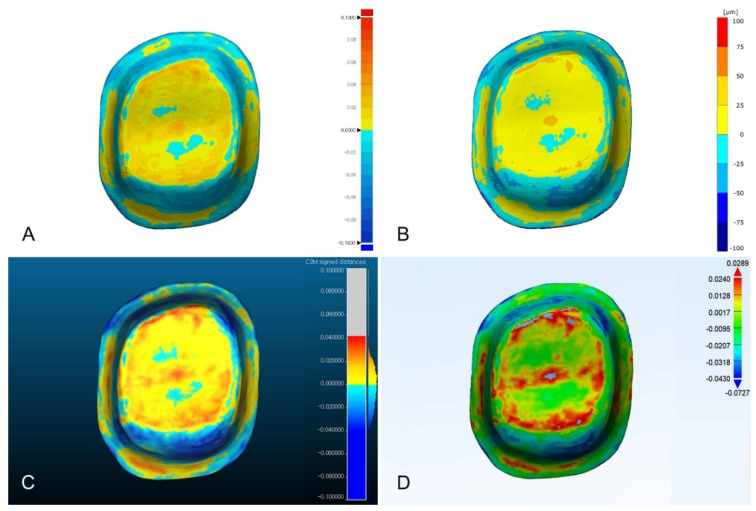
Comparison of color difference maps of tooth preparation region evaluated by using four analysis software programs: (**A**) Geomagic control X; (**B**) GOM Inspect; (**C**) Cloudcompare; (**D**) Materialise 3-matic.

Except for Materialise 3-matic, the distribution of color difference maps was similar (Figure 3, Figure 4, and Figure 5). In the color difference maps of complete arch, blue color regions were shown on the incisal regions of anterior teeth and lingual surfaces of molars, and red color regions were shown on the buccal surfaces of the posterior teeth (Figure 3). In the color difference maps of half arch, blue color regions were shown on the incisal regions of anterior teeth, and red color regions were shown on the occlusal surfaces of premolars and first molar (Figure 4). In the color difference maps of tooth preparation, blue color region was shown on the axial regions of the abutment, and red color regions were shown on the occlusal regions of the abutment (Figure 5).

**Figure 6:**
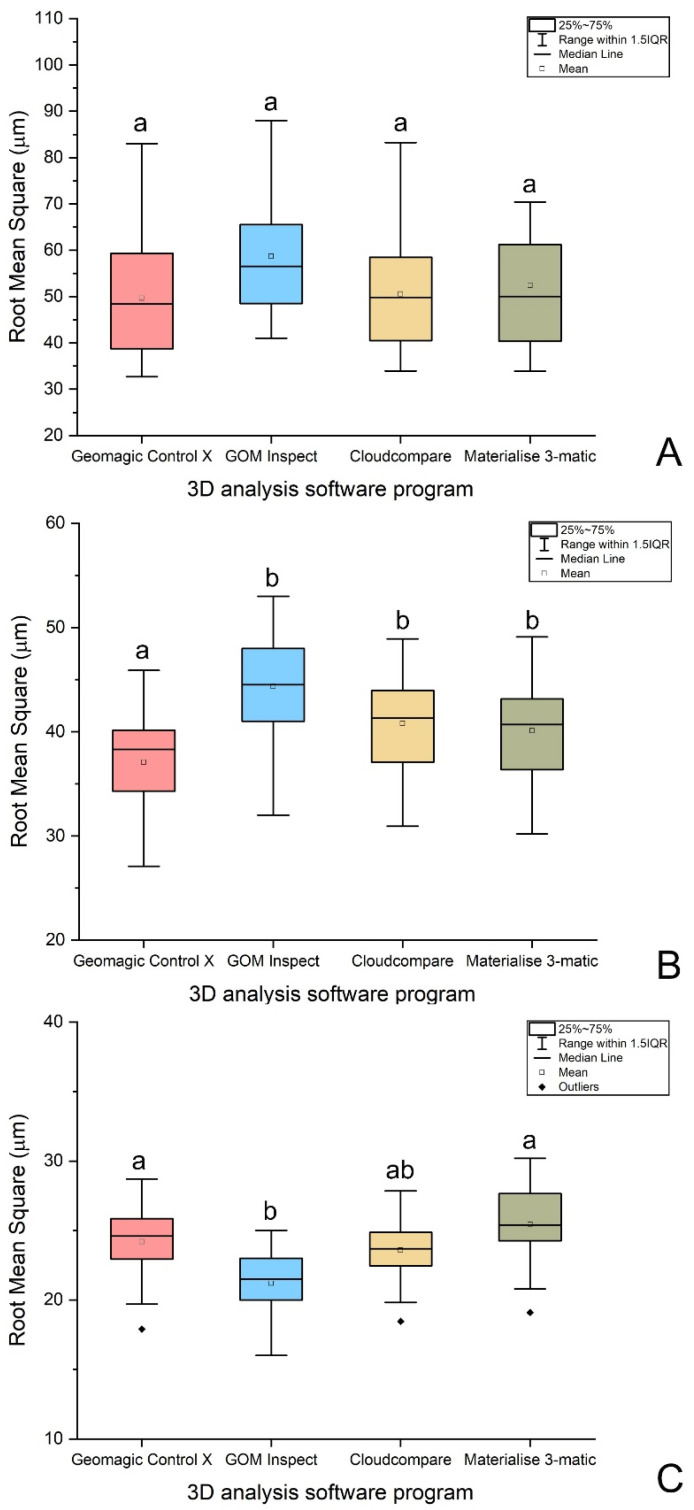
Comparison of root mean squares evaluated by using four analysis software programs. (**A**) Complete-arch region; (**B**) Half-arch region; (**C**) Tooth preparation region. The letters (a and b) indicate significant differences among software program groups using Tukey HSD test, *p* < 0.05.

In the four software programs, significant differences were found in the half arch (F = 6.893; *p* < 0.001) and tooth preparation (F = 10.211; *p* < 0.001) (Table 2, Figure 6). By contrast, in the complete arch, no significant difference was found among the four software programs (F = 1.888; *p* = 0.139; Table 2, Figure 6).

When different RMS calculation methods in the four software programs were applied, a significant difference was observed according to the software program (F = 4.291; *p* = 0.007; Table 3). Conversely, when different alignment procedures in the four software programs were applied, no significant difference was found according to the software program (F = 0.475; *p* = 0.701; Table 3).

A significant difference was found in the RMS values according to the software program (F = 3.022; *p* = 0.031) and the analysis area (F = 247.564; *p* < 0.001; Table 4). The software program and the analysis area had an interactive effect (F = 2.621; *p* = 0.018; Table 4).

## 4. Discussion

The present study analyzed the accuracy of complete arch, half arch, and tooth preparation by using four analysis software programs, and compared the differences among the software programs. The RMS values of half arch and tooth preparation were significantly different depending on the software program used (*p* < 0.001; Table 2), but no significant difference was found in the complete arch (*p* = 0.139; Table 2). Therefore, the null hypothesis was partially rejected. In addition, after the identical alignment method, the different RMS calculation methods in each software program had a significant effect on the RMS results (*p* = 0.007; Table 3), but the different alignment methods in each software program did not cause a significant difference in the RMS results (*p* = 0.701; Table 3). Therefore, the RMS calculation method may affect the RMS results more than the different alignment methods of the software programs.

Many studies have reported the use of various software programs for 3D analysis [7,22,23,24]. 3D analysis is sometimes applied to evaluate the processing precision of CAM as the surgical static guide for implant surgery and for the fixed and removable dental prosthesis fabricated by 3D printing or milling [1,12,13,26,27]; however, many studies have applied 3D analysis to evaluate the accuracy of 3D scanners [1,4,10,11]. The four software programs used in this study were used as evaluation tools for the accuracy of the 3D scanner in previous studies [2,6,15,16]. Of the four software programs, Geomagic control X has been used in most studies [3,14,26]. Materialise 3-matic is a 3D modeling software, and the 3D analysis applied in this study is an additional function of the software. In addition, GOM Inspect and Cloudcompare are free-to-use software, and Cloudcompare provides unlimited access to all its features. The results of this study reveal significant differences in the results depending on the software program used, but no difference was noted in the results for 3D analysis in a wide area such as in complete-arch region (Table 2); hence, the application of free software can be sufficiently considered.

No difference was observed in the RMS values for the different alignments performed in the four software programs (*p* = 0.701; Table 3). A study suggested that the distance between two objects can be minimized through the ICP algorithm in the alignment procedure [28]. Another study reported that the ICP algorithm can reduce errors in dental casts [21]. The ICP algorithm largely progressed in six stages: (1) selection of some sets of points in a range image, (2) matching these points to samples in the other meshes, (3) weighting the corresponding pairs appropriately, (4) rejecting certain pairs by observing each pair individually or considering the entire set of pairs, (5) assigning an error metric based on the point pairs, and (6) minimizing the error metric [30]. All software programs used in this study were optimized through the ICP algorithm. Although the calculation method of a specific ICP algorithm may differ depending on the software manufacturer [30], in this study, the alignment methods of the four software programs did not affect the RMS results.

**Figure 7:**
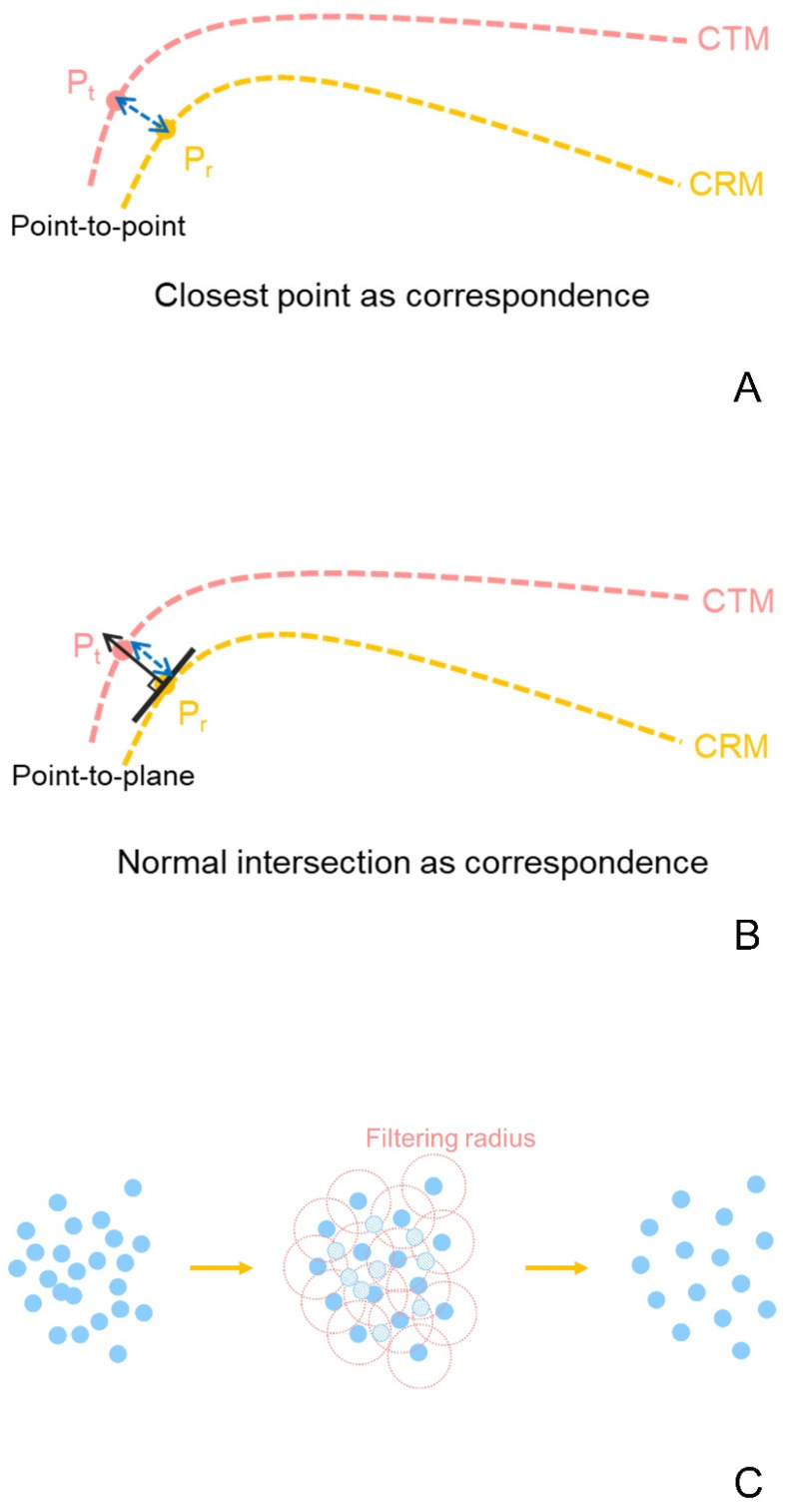
Schematic representation of corresponding point and point clouds’ sampling: (**A**) Point-to-point; (**B**) Point-to-plane; (**C**) Sampling of point clouds by filtering radius. CTM, CAD test model; CRM, CAD reference model.

In this study, when the identical alignment process and different RMS calculations of the four software programs were performed, the RMS values were different (*p* = 0.007; Table 3). In addition, a study reported that the RMS calculation may differ depending on the software applications and methods (cloud-to-cloud vs. cloud-to-mesh) [29]. The reason for the large difference in calculation is the heterogeneity of methods in selecting the corresponding point between the point cloud of CRM and CTM [29]. This can be divided into a method for obtaining the shortest distance between a point and another point (Figure 7A) [19] and a method for obtaining the shortest distance between a point and tangent plane (Figure 7B) [18]. This difference can have a great influence on the RMS value [18,19]. In addition, calculating for all point clouds takes a substantial amount of time because the algorithm for sampling among all points differs depending on the software program used (Figure 7C) [31]. Therefore, a difference can be seen in the calculated RMS value in each of the four software programs used in this study.

In this study, no difference was found in the RMS among software in the analysis for complete arch (*p* = 0.139; Table 2), but a difference was noted in the results among software as the analysis area became smaller (*p* < 0.001; Table 2). The results of this study reveal that the RMS calculation method affects the RMS result (Table 3), and according to a previous study, the results may vary depending on the sampling of point clouds (Figure 7C) [31]. Therefore, no difference in RMS among software programs in the complete-arch group was found because the area in which the RMS value can be calculated is wide; hence, the difference among software programs can be reduced. For this reason, when analyzing an area that is too small than the complete arch, the bias of the result should be considered.

3D analysis software was first developed in automotive and manufacturing industries for verifying printouts [20,25]. Recently, with the expansion of the dental digital workflow, studies have tried to verify the results of manufacturing and scan data [5,8,9]. Therefore, it is necessary to compare differences among software, as in this study. For future comparative studies, consensus on the use of a software program should be reached through additional standardized experiments.

In previous studies, in vivo experiments were performed to analyze the accuracy of intraoral scanners [32,33]. In the present study, since analysis software programs were used as variables, the accuracy of the intraoral scanner was evaluated in an in vitro environment. As it is difficult to obtain CRM that can be used as a more precise standard in the patient’s oral cavity, many previous studies have evaluated the accuracy of intraoral scanners in an in vitro environment [4,5,7,8]. However, in evaluating the accuracy of intraoral scanners for clinical applications, intraoral conditions (saliva, movement of the mandible and maxilla, and limited space due to limitation of mouth opening) must be taken into consideration [34,35,36]. Therefore, for the purpose of evaluating the accuracy of the intraoral scanner, additional in vivo experiments should be performed.

In the present study, three different analysis regions (complete arch, half arch, and tooth preparation) were compared. The reason for comparing the various analysis regions is that the range of intraoral scan varies according to the purpose for dental prosthetic treatment. Therefore, the region from the minimum range for the tooth preparation to the maximum range for the complete arch was analyzed. The complete-arch region showed the same results depending on the software program (*p* = 0.139), but the results for half arch and tooth preparation differed depending on the software program (*p* < 0.001). According to the results of one-way ANOVA, it can be considered that a larger F value means that there is a difference in variance between groups (Table 2). Therefore, it can be seen that tooth preparation (F = 10.211) showed a greater difference among groups according to the software program than half arch (F = 6.893).

This study has some limitations. Various setting conditions were excluded in the alignment and analysis process. However, this study used the default values recommended by each software program; if the user does not understand these settings, the resulting values may vary greatly. Therefore, the clinical validity of these differences should be verified through additional studies.

## 5. Conclusions

Within the limitations of this in vitro study, the following conclusions can be drawn:

- As the area of the virtual model for 3D analysis becomes smaller, a difference in results occurs depending on the software program used.
- The difference in results among software programs is not considered in the 3D analysis for the complete-arch region.
- Differences in the results are due to the heterogeneity of RMS calculation algorithms rather than on the different alignment algorithms of the software program used.
- Therefore, in light of these conclusions, the accuracy analysis of the intraoral scanner for complete arch can be evaluated without considering the software program.
- The use of software programs for 3D analysis should be determined according to the clinical situation.

## Figures and Tables

**Table 1:** Three-dimensional analysis software programs used.

3D Analysis Software Programs	Function						Version	Payment
	Alignment			3D Analysis				
	Initial Alignment	Optimal Alignment	Align Algorithm		3D Comparison	Calculation Principle		
Geomagic control X	Initial alignment	Best-fit alignment	Iterative closest point		3D compare	Root mean square	v2019.0.1	Paid
GOM Inspect	Pre alignment	Local best-fit	Iterative closest point		Surface comparison	Root mean square	v2.0.1	Free (Partially paid)
Cloudcompare	Point pairs picking	Fine registration	Iterative closest point		Mesh distance	Root mean square	v2.12	Free
Materialise 3-matic	N points registration	Global registration	Iterative closest point		Part comparison analysis	Root mean square	v13.0	Paid

**Table 2:** Comparison of RMS (µm) of complete-arch, half-arch, and tooth preparation region evaluated by using four analysis software programs.

Analysis Region	Descriptive Statistics		3D Analysis Software Program				F	*p* *
			Geomagic Control X	GOM Inspect	Cloudcompare	Materialise 3-Matic		
Complete arch	Mean ± SD		49.6 ± 13.0	58.7 ± 12.6	50.3 ± 12.1	52.4 ± 15.1	1.888	0.139
	95% CI	Lower	43.5	52.7	44.8	45.3		
		Upper	55.7	64.6	56.2	59.5		
Half arch	Mean ± SD		37.0 ± 5.1 ^a^	44.3 ± 5.4 ^b^	40.8 ± 4.8 ^b^	40.0 ± 4.8 ^b^	6.893	<0.001
	95% CI	Lower	34.7	41.7	38.5	37.8		
		Upper	39.4	46.9	43.0	42.3		
Tooth preparation	Mean ± SD		24.1 ± 2.5 ^a^	21.2 ± 2.2 ^b^	23.6 ± 2.2 ^a,b^	25.4 ± 2.8 ^b^	10.211	<0.001
	95% CI	Lower	22.9	20.1	22.5	24.1		
		Upper	25.1	22.2	24.6	26.7		

* Significance determined using one-way ANOVA, *p* < 0.05. Different letters ^a, b^ indicate significant differences among software program groups using Tukey HSD test, *p* < 0.05. CI, confidence interval; RMS, root mean square; SD, standard deviation.

**Table 3:** Comparison of RMS (µm) of tooth preparation region evaluated by using an identical alignment procedure and RMS calculation.

	Descriptive Statistics		3D Analysis Software Program				F	*p* *
			Geomagic Control X	GOM Inspect	Cloudcompare	Materialise 3-Matic		
Different RMS calculation methods	Mean ± SD		24.1 ± 2.5 ^a,b^	23.1 ± 1.9 ^a^	24.6 ± 1.9 ^a,b^	25.5 ± 2.2 ^b^	4.291	0.007
	95% CI	Lower	17.9	19.0	20.3	20.7		
		Upper	28.7	26.0	28.4	28.9		
Different alignment procedure	Mean ± SD		24.1 ± 2.5	24.2 ± 2.5	24.2 ± 2.5	25.0 ± 3.1	0.475	0.701
	95% CI	Lower	17.9	17.9	18.0	18.0		
		Upper	28.7	28.7	28.8	30.2		

* Significance determined using one-way ANOVA, *p* < 0.05. Different letters ^a, b^ indicate significant differences among software program groups using Tukey HSD test, *p* < 0.05. CI, confidence interval; RMS, root mean square; SD, standard deviation.

**Table 4:** Results of the ANOVA of the three-dimensional analysis software program and analysis region.

Source	F	*p*
3D analysis software program	3.022	0.031 *
Analysis region	247.564	<0.001 *
3D analysis software program x Analysis region	2.621	0.018 **

ANOVA, analysis of variance. Significance determined by * one-way ANOVA and ** two-way ANOVA, *p* < 0.05.

## Data Availability

Data is contained within the article.

## Abbreviations

CAD/CAM	computer-aided design and computer-aided manufacturing
3D	three-dimensional
CRM	computer-aided design reference model
CTM	computer-aided design test model
ICP	iterative closest point
RMS	root mean square
ANOVA	analysis of variance
HSD	honestly significant difference
